# JNK-mediated Slit-Robo signaling facilitates epithelial wound repair by extruding dying cells

**DOI:** 10.1038/s41598-019-56137-z

**Published:** 2019-12-20

**Authors:** Chiaki Iida, Shizue Ohsawa, Kiichiro Taniguchi, Masatoshi Yamamoto, Ginés Morata, Tatsushi Igaki

**Affiliations:** 1 Laboratory of Genetics, Graduate School of Biostudies, Kyoto University, Yoshida-Konoe-cho, Sakyo-ku, Kyoto, Kyoto, 606-8501 Japan; 2 Group of Genetics, Division of Biological Science, Graduate School of Science, Nagoya University, Furo-cho, Chikusa-ku, Nagoya, Aichi 464-8602 Japan; 3 Centro de Biología Molecular, CSIC-UAM, Universidad Autónoma de Madrid, 1, Nicolás Cabrera, Madrid, 28049 Spain; 4 Present Address: Department of Cancer Biology, Graduate School of Medical Sciences, Kumamoto University, Honjo, Chuo-ku, Kumamoto, 860-8556 Japan

**Keywords:** Extracellular signalling molecules, Stress signalling

## Abstract

Multicellular organisms repair injured epithelium by evolutionarily conserved biological processes including activation of c-Jun N-terminal kinase (JNK) signaling. Here, we show in *Drosophila* imaginal epithelium that physical injury leads to the emergence of dying cells, which are extruded from the wounded tissue by JNK-induced Slit-Roundabout2 (Robo2) repulsive signaling. Reducing Slit-Robo2 signaling in the wounded tissue suppresses extrusion of dying cells and generates aberrant cells with highly upregulated growth factors Wingless (Wg) and Decapentaplegic (Dpp). The inappropriately elevated Wg and Dpp impairs wound repair, as halving one of these growth factor genes cancelled wound healing defects caused by Slit-Robo2 downregulation. Our data suggest that JNK-mediated Slit-Robo2 signaling contributes to epithelial wound repair by promoting extrusion of dying cells from the wounded tissue, which facilitates transient and appropriate induction of growth factors for proper wound healing.

## Introduction

Wound repair is an evolutionarily conserved process that maintains tissue homeostasis upon injury^1–3^. It has been reported that JNK signaling acts as an essential regulator of wound repair in *Drosophila* epithelial tissue^4–7^, planarians body^8,9^, and zebrafish tail fin^10^. Genetic studies in *Drosophila* have shown that JNK signaling contributes to (1) actin remodeling to close wound edges^6,11^, (2) reconstruction of lost tissue parts by activating growth promoters such as Yorkie (Yki, a YAP homolog)^12,13^, Wg (a Wnt homolog)^14^, Dpp (a TGF-β/BMP family member)^15^ and Myc^14^, (3) facilitating cell reprograming via reducing the activity of polycomb-dependent silencing^16^, and (4) induction of developmental delay by upregulating *Drosophila* insulin-like peptide 8 (Dilp8) to prolong the developmental period for recovery^17^. Particularly, JNK-dependent induction of Wg promotes regenerative growth of *Drosophila* wing imaginal discs after genetic ablation of the tissue^14^. In addition, JNK-mediated upregulation of Wg and Dpp plays critical roles in compensatory proliferation of imaginal cells after induction of massive cell death^15,18,19^. JNK signaling also induces apoptosis^20,21^, which is required for regeneration of planarian body^9^ or wound repair in *Drosophila* epithelial tissue^22–24^. Together, JNK regulates multiple steps of repair process from beginning to end.

Dying cells emerged in the epithelial tissue are extruded basally or apically by a coordinated mechanism^25^. For instance, overcrowding of cells within a limited space triggers extrusion of living or dying cells from Madin-Darby canine kidney (MDCK) epithelial monolayer^26^, developing zebrafish tail fin^26^, and *Drosophila* notum^27^. In *Drosophila* embryonic development, extrusion of apoptotic cells from amnioserosa promotes dorsal closure^28,29^, the process that shares common JNK-dependent events with epithelial wound repair, which include actin remodeling, cell migration, and epithelial zipping^30,31^. Similarly, JNK-dependent cell extrusion is required for tumor-suppressive cell competition, the process in which oncogenic polarity-deficient cells such as *scribble* (*scrib*) or *discs large* (*dlg*) mutant cells are actively eliminated from epithelia when surrounded by wild-type cells^32–37^. Importantly, extrusion of polarity-deficient cells by cell competition is driven by JNK-mediated activation of Slit-Robo2 axonal repulsive signaling that downregulates E-cadherin, as the ligand Slit, its receptor Robo2, and the downstream effector Enabled (Ena)/Vasp are all induced by JNK signaling^35^. During *Drosophila* embryonic development, the N-terminus of Slit produced from midline glial cells binds to the immunoglobulin (Ig) motif of Robo2 expressed in commissural axons, thereby regulating midline crossing of commissural axons via cell-cell repulsion^38–43^ and the system is well conserved throughout evolution^44^. Interestingly, it has been shown that extrusion of dying cells by Semaphorin-PlexinA axonal repulsive signaling is required for wound repair in *Drosophila* and zebrafish epithelia^45^, although the role of cell extrusion in wound repair and the upstream trigger for cell-extrusion signaling remain unknown.

Here, we found in *Drosophila* epithelium that physical injury induces JNK activation, which promotes extrusion of dying cells via Slit-Robo2 signaling. The Slit-Robo2-mediated cell extrusion facilitates epithelial wound repair by preventing excessive expression of growth factors Wg and Dpp upon injury.

## Results and Discussion

### Slit-Robo2 signaling acts downstream of JNK in wound repair

**Figure 1:**
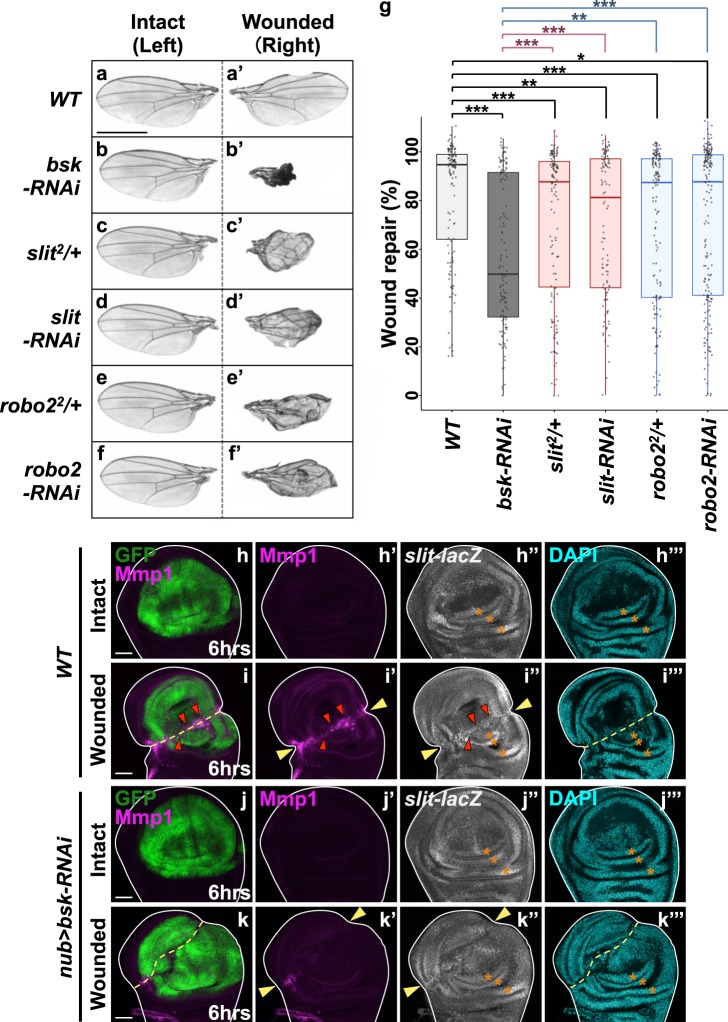
Slit-Robo2 signaling acts downstream of JNK in wound repair. (**a**–**f**’) Control intact (**a**–**f**) and wounded (**a**’–**f**’) adult wings in each genotype taken from the same individuals. All pictures were taken at the same magnification. Scale bar, 500 µm. (**g**) Boxplot with dots representing wound repair (%) (see Methods) in each genotype (wild-type (n = 136), *bsk-RNAi* (n = 131), *slit*^2^/+ (n = 139), *slit-RNAi* (n = 113), *robo*2^2^/+ (n = 154), and *robo*2*-RNAi* (n = 189)). Mann-Whitney *U*-test; *p < 0.05, **p < 0.01, and ***p < 0.001. (**h**–**k**”’) Wing discs of wild-type (**h**–**i**”’) and *nub* > *bsk-RNAi* (**j**–**k**”’) flies were dissected at 6hrs after wounding. Wing pouches were labeled with GFP using the *nub-gal4* driver (green). JNK activity (magenta), *slit-lacZ* expression (white), and nuclei (cyan) were detected by anti-Mmp1, anti-β-gal (for *slit-lacZ*), and DAPI, respectively. Yellow dashed lines and arrowheads indicate locations of wounds. Red arrowheads indicate ectopic *slit-lacZ* expressions. Asterisks indicate the position of endogenous *slit-lacZ* expression. Scale bars, 50 µm. See Supplementary Information for detailed genotypes.

To dissect the mechanism of epithelial wound repair in *Drosophila*, we physically injured the wing imaginal disc, the larval epithelial primordia of adult wing. The right wing disc was injured with a tungsten needle by aseptic *in situ* wounding in living larvae without further damaging the animal (hereafter denoted as “wounded” disc), with the left wing disc remained undamaged as an internal control (hereafter denoted as “intact” disc) (Supplementary Fig. 1). Wounded wing discs were repaired during animal development and form essentially normal adult wings (Fig. 1a,a’, quantified in Fig. 1g). Blocking JNK signaling by knocking down *Drosophila* JNK *basket* (*bsk*) significantly impaired wound repair (Fig. 1b,b’, quantified in Fig. 1g), indicating that JNK signaling is essential for wound repair as reported previously^4–7^. In analyzing downstream effectors of JNK signaling, we found that Slit-Robo2 signaling, the cell-extrusion signaling activated by JNK during tumor-suppressive cell competition^35^, is required for wound repair. Downregulation of Slit or Robo2 by heterozygous deletion of these genes or by RNAi expression in the wing pouch significantly impaired wound repair (Fig. 1c’–f’, quantified in Fig. 1g) without affecting wing development in intact discs (Fig. 1c–f). Consistently, JNK activity and *slit-lacZ* expression were elevated around the wound at 6hrs after wounding, as visualized by the anti-Mmp1^46^ and *slit-lacZ* reporter (Fig. 1h,i). The upregulations of Mmp1 and *slit-lacZ* expression were significantly suppressed by *bsk*-RNAi (Fig. 1j–k”, quantified in Supplementary Fig. 2). In addition, blocking Slit-Robo2 signaling did not exacerbate repair defect caused by *bsk-*RNAi (Supplementary Fig. 3a,b, quantified in Supplementary Fig. 3c), suggesting that JNK and Slit-Robo2 participate in wound healing process in the same pathway. These data suggest that Slit-Robo2 signaling acts downstream of JNK in wound repair. The wound-repair defect caused by blocking JNK was severer than blocking Slit-Robo2 (Fig. 1g), likely because JNK has multiple functions in wound repair.

### Slit-Robo2 signaling promotes extrusion of dying cells from the wounded tissue

**Figure 2:**
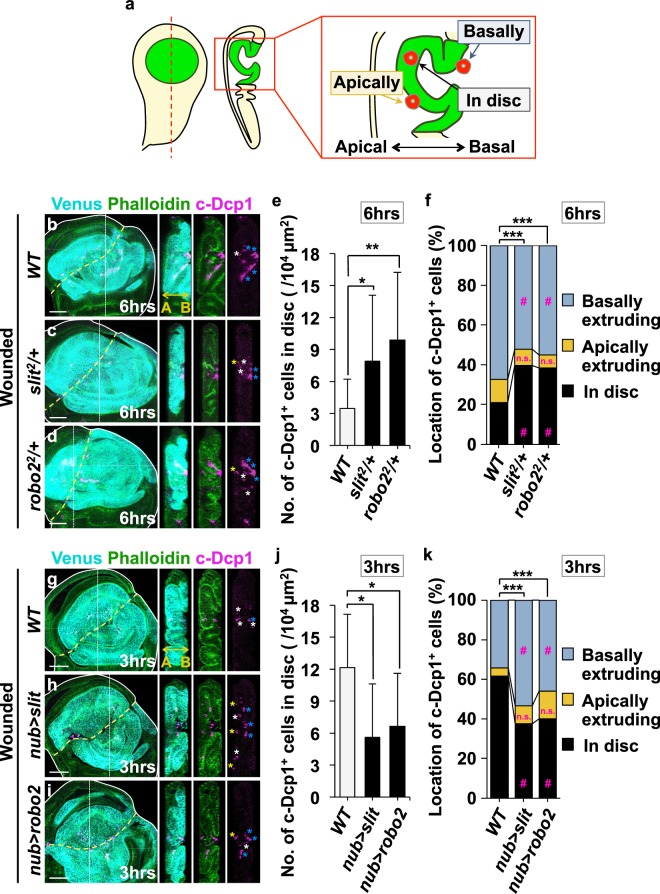
Slit-Robo2 promotes extrusion of dying cells from the wounded tissue. (**a**) Diagram of the analysis for dying cell locations in the cross-section of the wing disc. See Methods for further details. (**b**–**k**) Images show *xy* and *yz* cross-sections of wounded wing discs of wild-type (**b**), *slit*^2^/+ (**c**), and *robo*2^2^/+ (**d**) larvae dissected at 6hrs after wounding; *xy* and *yz* cross-sections of wild-type (**g**), *nub* > *slit* (**h**), and *nub* > *robo*2 (**i**) larvae dissected at 3hrs after wounding. Dying cells were detected by anti-c-Dcp1 staining (magenta) and wing pouches were marked with Venus using the *nub-gal4* driver (cyan), and F-actin was visualized with Phalloidin (green). Yellow dashed lines indicate the positions of wounds. White dashed lines indicate the positions of *yz* cross-section shown in the right panel. The two-direction arrow indicates apical (A) and basal (B) sides of the disc. Asterisks in right panels indicate dying cells classified as “in disc” (white), “apically extruding” (yellow), and “basally extruding” (blue). Scale bars, 50 µm. Quantification of the number of dying cells classified as “in disc” at 6hrs after wounding in each genotype (**e**) (wild-type (n = 12), *slit*^2^/+ (n = 12), and *robo*2^2^/+ (n = 12)); 3hrs after wounding in each genotype (**j**) (wild-type (n = 11), *nub* > *slit* (n = 12), and *nub* > *robo*2 (n = 12)). Welch’s T-test; mean ± s.d.; *p < 0.05 and **p < 0.01. (**f**,**k**) Quantification of the ratio of dying cells classified into 3 types (as shown in A) at 6hrs after wounding in each genotype (**f**) (wild-type (n = 12), *slit*^2^/+ (n = 12), and *robo*2^*2*^/+ (n = 14)); 3hrs after wounding in each genotype (**k**) (wild-type (n = 17), *nub* > *slit* (n = 13), and *nub* > *robo2* (n = 13)). Chi-squared test; ***p < 0.001; # the absolute value of adjusted residual >2.56. See Supplementary Information for detailed genotypes.

Our finding that Slit-Robo2 signaling plays a role in epithelial wound repair suggests that JNK-mediated cell extrusion is required for this process. We thus analyzed spatial locations of dying cells in the wounded tissue by immunostaining for the cleaved form of the effector caspase Dcp1 (c-Dcp1). In wild-type background, the number of dying cells in the wing pouch significantly increased at 6hrs after wounding (Supplementary Fig. 4a,b, quantified in Supplementary Fig. 4i). Importantly, the number of dying cells within the disc was 4-fold higher at the earlier time point (Fig. 2g, 3hrs, quantified in Fig. 2j, compare to Fig. 2b, 6hrs, quantified in Fig. 2e), suggesting that dying cells are extruded from the tissue over time. Supporting this notion, the analysis of extruding/extruded dying cells in the wounded discs by classifying their locations into three classes (“in disc”, “apically extruding”, and “basally extruding”; Fig. 2a) revealed that the ratio of dying cells within the disc over basally/apically extruding cells was significantly reduced over time (compare Fig. 2k (3 hrs, wild-type), Fig. 2f (6 hrs, wild-type), and Supplementary Fig. 5e,l (9 hrs, wild-type)). Crucially, heterozygous deletion of *slit* or *robo*2 gene significantly increased the number and ratio of dying cells remained in the wounded disc at 6hrs (Fig. 2c,d, quantified in Fig. 2e,f) and 9hrs (Supplementary Fig. 5f,g, quantified in Supplementary Fig. 5l) after wounding, while the tendency was not observed at 3hrs after wounding likely because extrusion has not proceeded sufficiently at this time point even in the wild-type tissue (Supplementary Fig. 5a,b, quantified in Supplementary Fig. 5j).

Conversely, overexpression of *slit* or *robo*2 in the wing pouch significantly decreased the number and ratio of dying cells in the disc at 3hrs (Fig. 2h,i, quantified in 2j, k) and 6hrs (Supplementary Fig. 5c,d, quantified in Supplementary Fig. 5k) after wounding, while the tendency was not observed at 9hrs after wounding likely because most cells have already been extruded by this time point even in the wild-type tissue (Supplementary Fig. 5h,i, quantified in Supplementary Fig. 5l). Together, these data indicate that JNK-induced Slit-Robo2 signaling promotes extrusion of dying cells from the injured tissue, which is essential for wound repair. Notably, blocking JNK signaling in the damaged wing pouch abolished apoptosis (as visualized by c-Dcp1 staining) but not necrosis (as visualized by Propidium Iodide (PI) staining) (Supplementary Fig. 4), indicating that apoptosis induction is also JNK-dependent as reported previously^22–24^.

### Slit-Robo2-mediated extrusion of dying cells prevents excessive growth factor expressions in the wounded tissue

**Figure 3:**
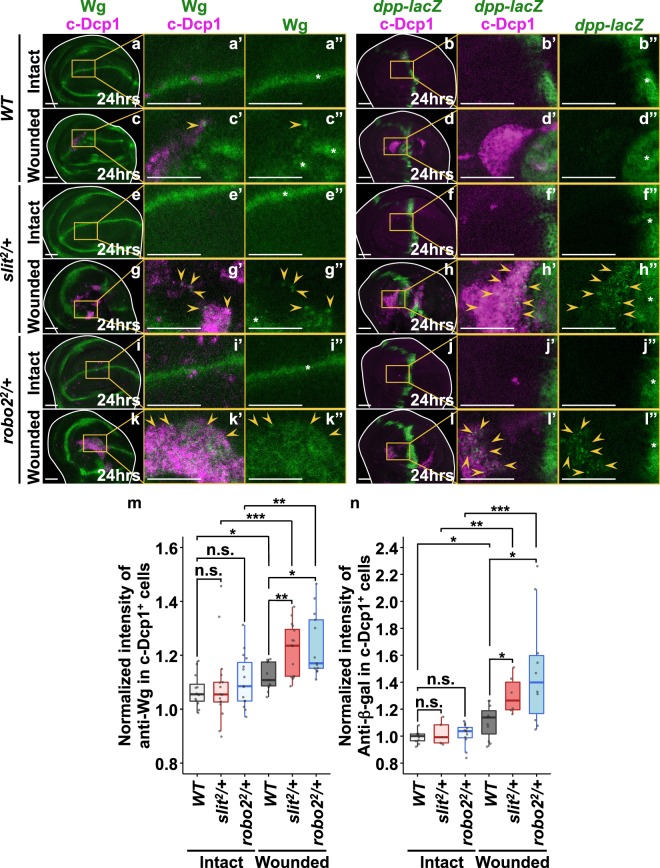
Defects in dying cell extrusion cause aberrant Wg and Dpp expression. (**a**–**l**”) *xy* cross-section images of intact and wounded wing discs of wild-type (**a**–**d**”), *slit*^*2*^/+ (**e**–**h**”), and *robo2*^*2*^/+ (**i**–**l**”) larvae at 24hrs after wounding. Dying cells were detected with anti-c-Dcp1 antibody (magenta). Wg and Dpp expressions were detected using anti-Wg antibody and anti-β-gal antibody (for *dpp-lacZ*), respectively (green). Yellow arrowheads indicate representative dying cells expressing aberrantly high levels of Wg or Dpp. Asterisks indicate endogenous signals. Scale bars, 50 µm. (**m**,**n**) Boxplot with dots representing normalized intensity of anti-Wg in c-Dcp1-positive cells (see Methods) in each genotype (wild-type (intact: n = 12, wounded: n = 11), *slit*^*2*^/+ (intact: n = 16, wounded: n = 17), and *robo2*^*2*^/+ (intact: n = 15, wounded: n = 13)). (**m**) Boxplot with dots representing normalized intensity of anti-β-gal antibody (for *dpp-lacZ*) in c-Dcp1-positive cells (see Methods) at 24hrs after wounding in each genotype (wild-type (intact: n = 9, wounded: n = 11), *slit*^*2*^/+ (intact: n = 5, wounded: n = 6), and *robo2*^*2*^/+ (intact: n = 12, wounded: n = 10)). (**n**) Mann-Whitney *U*-test; *p < 0.05, **p < 0.01, ***p < 0.001; n.s.; not significant. See Supplementary Information for detailed genotypes.

**Figure 4:**
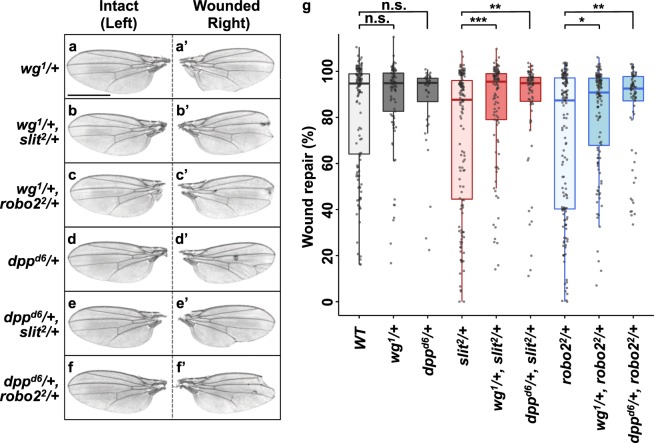
w*g* and *dpp* mutants could rescue Slit-Robo2 defect. (**a**–**f**’) Control intact (**a**–**f**) and wounded (**a**’–**f**’) adult wings in each genotype taken from the same individuals after wounding. All pictures were taken at the same magnification. Scale bar, 500 µm. (**g**) Boxplot with dots representing wound repair (%) (see Methods for details) in each genotype (wild-type (n = 138), *wg*^1^/+ (n = 89), *dpp*^*d6*^/+ (n = 64), *slit*^2^/+ (n = 139), *slit*^2^/+*, wg*^1^/+ (n = 105), *slit*^2^/+*, dpp*^*d6*^/+ (n = 77), *robo*2^2^/+ (n = 154), *robo2*^*2*^/+*, wg*^1^/+ (n = 128), and *robo*2^*2*^/+*, dpp*^*d6*^/+ (n = 95)) Mann-Whitney *U*-test; *p < 0.05, **p < 0.01, ***p < 0.001; n.s.; not significant. See Supplementary Information for detailed genotypes.

**Figure 5:**
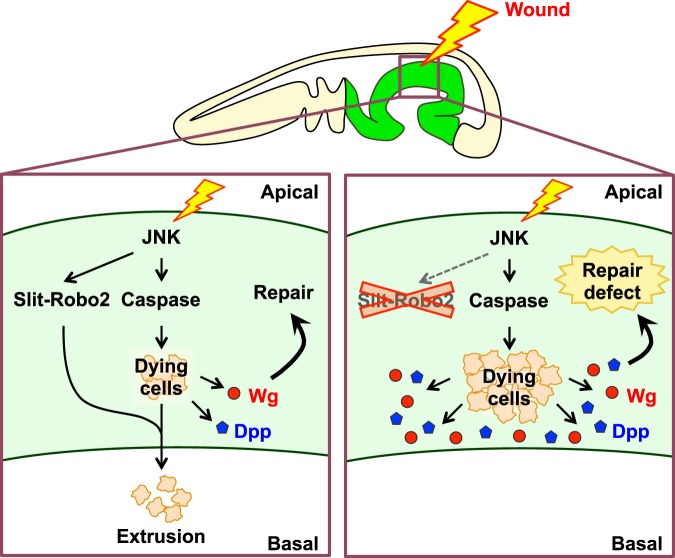
Model for the role of JNK-Slit-Robo-mediated extrusion of dying cells in wound repair. See text for details.

We next examined the role of Slit-Robo2-mediated extrusion of dying cells in wound repair. It has been shown that secreted growth factors Wg and Dpp are essential for regeneration of damaged epithelia in *Drosophila*^14,15,18,19,47^. Interestingly, we found that at 24hrs after wounding, aberrant dying cells with highly elevated Wg and Dpp expressions were emerged in *slit*^*2*^/+ or *robo*2^*2*^/+ wounded wing discs (Fig. 3g,h,k,l) compared to wild-type background (Fig. 3c,d, quantified in Fig. 3m,n), while intact discs did not possess such aberrant cells (Fig. 3a,b,e,f,i,j, quantified in Fig. 3m,n). In addition, these aberrant cells frequently located nearby or within JNK-activated cells as visualized by *TRE-DsRed* reporter^48^ (Supplementary Fig. 6g (63.6%, n = 11), h (100%, n = 5), k (85.7%, n = 14), l (87.5%, n = 14)) compared to wild-type background (Supplementary Fig. 6c (30.8%, n = 13), d (22.2%, n = 9)). These data indicate that impaired Slit-Robo2 signaling in the wounded tissue results in the emergence of abnormal cells with excess production of Wg and Dpp, which may disturb the wound healing process. Indeed, downergulation of Wg or Dpp by halving the *wg* or *dpp* gene significantly suppressed the wound healing defects in *slit*^*2*^/+ or *robo2*^*2*^/+ flies (Fig. 4b,c,e,f, quantified in Fig. 4g, and Supplementary Fig. 7b,c,e,f, h,i, quantified in Supplementary Fig. 7j), while heterozygosity for *wg* or *dpp* on its own did not affect wound repair (Fig. 4a,d, quantified in Fig. 4g, and Supplementary Fig. 7a,d,g, quantified in Supplementary Fig. 7j). In addition, halving the *wg* gene indeed suppressed the emergence of aberrant cells with excess Wg (Supplementary Fig. 8a–f, quantified in Supplementary Fig. 8g). These results suggest that the emergence of aberrant cells with excess growth factors is responsible for the repair defect. Together, our data indicate that JNK-induced Slit-Robo2 signaling contributes to wound repair by promoting extrusion of dying cells with excess growth factors (Fig. 5).

Intriguingly, it has been reported that an axon guidance molecule PlexinA plays an important role in cell extrusion during epithelial wound repair in *Drosophila* and zebrafish^45^. In addition, Slit has been proposed to bind to PlexinA in mammals^49^, suggesting that multiple axon guidance signaling contribute to wound healing by promoting dying cell extrusion. Our findings suggest that dying cells remained in the tissue with excess growth factors need to be removed for proper wound healing, by promoting epithelial fusion and/or facilitating transient and appropriate production of growth factors.

## Methods

### Fly strains

Flies were cultured with standard food in plastic vials at 25 °C. 3^rd^ instar wandering larvae were analyzed in all the experiments. Fly strains are used as follows: *nub-gal4* (Bloomington Drosophila Stock Center [BDSC] #42699), *rn-gal4*^*GAL4-5*^ (BDSC #7405), *UAS-CD8-PARP-Venus* (gift from Yasushi Hiromi)^50^, *UAS-bsk-RNAi* (National Institute of Genetics [NIG] #5680R-2), *slit*^2^ (Drosophila Genomics and Genetic Resources [DGGR] #106948)^51^, *UAS-slit-RNAi* (BDSC #31468), *robo*2^*2*^ (DGGR #106843)^42^, *UAS-robo2-RNAi* (BDSC #34589), *slit*^05248^ (*slit-lacZ*, BDSC #12189)^52,53^*, UAS-slit* (gift from Tom Kidd), *UAS-robo2-HA* (gift from Talia Volk)*, wg*^1^ (BDSC #2978)^54^*, wg*^*SP-1*^ (BDSC #405)^55,56^, *dpp*^*d6*^ (DGGR #106644)^57^, *dpp*^*s11*^ (DGGR #106646)^57^, *dpp*^*hr92*^ (DGGR #106649)^58^, *TRE-DsRed* (BDSC #59012)^48^, *P{PZ}dpp*^10638^ (*dpp-lacZ*, BDSC #12379)^52^.

### Physical *in situ* wounding

3^rd^ instar wandering larvae were randomly collected and anesthetized with ice-water for around 10 minutes. Then their wing discs (which were marked by fluorescent proteins GFP or Venus) were injured on ice with a sharpened 0.3 mm tungsten needle by performing aseptic *in situ* wounding (by pushing the wing pouch region using the needle) in living larvae without further damaging the animal. Wounding was performed under the fluorescence binocular microscope. After wounding, larvae were cultured in fresh food vials and kept at 25 °C again. Late 3^rd^ instar larvae before wondering were wounded only when we analyzed wing discs 24 hrs after wounding. See Supplementary Fig. 1 for further information.

### Histology

Larval tissues were fixed and stained using standard immunohistochemical methods with rabbit anti-cleaved-Dcp1 (1:100, Cell Signaling Technology), chicken anti-β-galactosidase (1:2500, abcam), mouse anti-Mmp1 (1:100, from cocktail of 3A6B4, 3B8D12 and 5H7B11, Developmental Studies Hybridoma Bank [DSHB]), mouse anti-Wingless (1:100, DSHB), anti-mouse Alexa 546, 647 (1:250, Molecular Probes), anti-rabbit Alexa 546, 647 (1:250, Molecular Probes), anti-chicken Alexa 647 (1:250, Molecular Probes), and were mounted with 49,6-diamidino-2-phenylindole (DAPI)-containing SlowFade Gold Antifade Reagent (Molecular Probes). For detecting necrotic cells, larval tissues were dissected in Schnieder’s *Drosophila* medium containing 5% fetal bovine serum (FBS) and were immediately moved into fresh medium containing Propidium Iodide (PI; 1:1000, Wako Pure Chemical Industries), then tissues were analyzed after three times washing with phosphate buffered saline (PBS). Images were taken with Leica TCS SP8 confocal microscopes with Leica Application Suite X ver. 2.0.1.14392 (Leica Microsystems).

### Measurement of wing size

The right and left wings of adult flies were mounted on slide glasses. Leica binocular stereo microscope with LEICA FIRECAM ver. 3.4 (Leica Microsystems) was used to take pictures of the wings and whole bodies of adult flies. The wing size was automatically measured by Fiji ver. 2.0.0-rc-49/1.51k (https://imagej.net/Fiji). “Wing repair (%)” was defined as injured right wing area divided by intact left wing area (%), calculated by Microsoft Excel for Mac ver. 16.16.4 (Microsoft).

### Analysis of dying cells

Apoptotic dying cells were detected by c-Dcp1 antibody. For the analysis of spatial locations of c-Dcp1-positive dying cells, the locations were classified into 3 classes: (1) “basally extruding”, as dying cells located at the basal tip of the disc proper, (2) “apically extruding”, as dying cells located at the apical tip of the disc proper, and (3) “in disc”, as dying cells located within the disc proper (see Fig. 2a for further information). Wing discs with wound that crosses center of the wing pouches were analyzed for special locations of c-Dcp1-positive cells. c-Dcp1-positive cells classified as “in disc” were manually counted using *xz* or *yz* cross-section images, and pouch areas were manually measured with Fiji and calculated with Microsoft Excel for Mac. For the analysis of total number of c-Dcp1-positive cells in the pouch, the number of c-Dcp1-positive cells in the wing pouch and the size of the wing pouch areas were automatically counted using Z-stacked images with Fiji and calculated with Microsoft Excel for Mac. For the analysis of necrotic dying cells detected by PI staining, the number of PI-positive cells in the wing pouch were automatically counted using single *xy* cross-section images with Fiji and calculated with Microsoft Excel for Mac.

### Quantifications of signal intensities

To analyze expression levels of *slit-lacZ* or Mmp1, the signal intensity of anti-β-gal or anti-Mmp1 staining at the wounded area was measured and normalized with background intensity in the intact notum area. For the analysis of Wg or Dpp expression by anti-Wg or anti-β-gal antibody, the signal intensity in c-Dcp1-positive cells (other than endogenous Wg and Dpp) was measured and normalized with background intensity in the intact pouch area. All measurements were performed with Fiji and calculated with Microsoft Excel for Mac.

### Statistical analysis

All experiments were repeated at least three times. Mann-Whitney non-parametric test was used for analyzing adult wing sizes and signal intensity, Welch’s T-test was used for analyzing the number of dying cells, and chi-squared test was used for analyzing spatial locations of dying cells. Error bars in all bar graphs indicate standard deviation (s.d.). n.s.; not significant indicates p ≧ 0.05 or the absolute value of adjusted residual ≦2.56. All bar graphs and stacked graphs were prepared with Microsoft Excel for Mac. All boxplot graphs include the data of all individuals as dots and were prepared with R ver. 3.2.3 (https://www.r-project.org).

## Supplementary information

Supplementary Information
