# Supplementary material for: JNK-mediated Slit-Robo signaling facilitates epithelial wound repair by extruding dying cells
Source: Sci Rep. 2019 Dec 20;9:19549. doi: 10.1038/s41598-019-56137-z (PMC6925126; doi:10.1038/s41598-019-56137-z)
Supplement: Supplementary file 1 — Supplementary Information [file 41598_2019_56137_MOESM1_ESM.pdf]

Supplementary Information

JNK-mediated Slit-Robo signaling facilitates epithelial wound repair by extruding dying cells

Chiaki Iida[1], Shizue Ohsawa[1, 2], Kiichiro Taniguchi[1], Masatoshi Yamamoto[1, #], Ginés Morata[3] and Tatsushi Igaki[1*]

[1] Laboratory of Genetics, Graduate School of Biostudies, Kyoto University, Yoshida-Konoe-cho, Sakyo-ku, Kyoto, Kyoto 606-8501, Japan

[2] Group of Genetics, Division of Biological Science, Graduate School of Science, Nagoya University, Furo-cho, Chikusa-ku, Nagoya, Aichi 464-8602, Japan

[3] Centro de Biología Molecular, CSIC-UAM, Universidad Autónoma de Madrid, 1, Nicolás Cabrera, Madrid 28049, Spain

[#] Present address: Department of Cancer Biology, Graduate School of Medical Sciences, Kumamoto University, Honjo, Chuo-ku, Kumamoto 860-8556, Japan

[*] To whom correspondence should be addressed.

Tatsushi Igaki, Ph.D.

Email: igaki@lif.kyoto-u.ac.jp

TEL.: +81-75-753-7684

FAX: +81-75-753-7686

Supplementary Figure 1

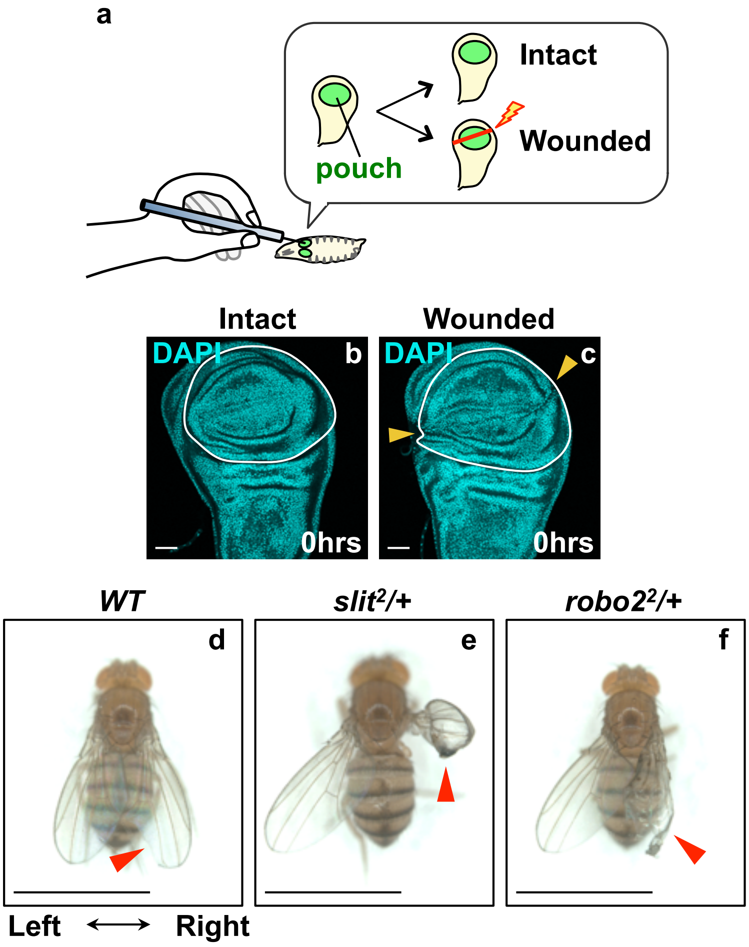

Aseptic *in situ* wounding in living larvae.

(a) The wing pouch of the right wing disc, which is labeled with GFP or Venus, was wounded with a sharpened tungsten needle on ice. (b, c) Intact (b) and wounded (c) wing discs dissected right after wounding. Yellow arrowheads indicate the position of the wound. White circle indicates the wing pouch. Scale bars, 50µm. (d-f) Whole-body adult pictures after *in situ* wounding of wild-type (d), *slit*^{2}/+ (e), and *robo2*^{2}/+ (f). Red arrowheads indicate the wounded right wings. Scale bars, 1 mm.

Supplementary Figure 2

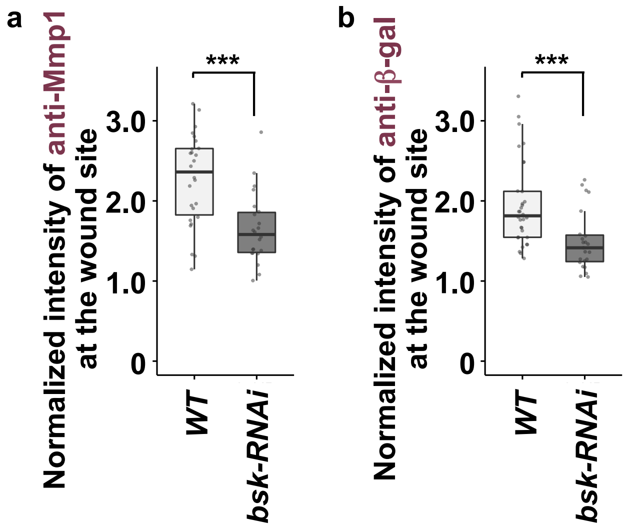

The upregulations of Mmp1 and *slit-lacZ* expression were significantly suppressed by *bsk*-RNAi.

(a, b) Boxplot with dots representing normalized intensity of anti-Mmp1 (a) and anti-β-gal (for *slit-lacZ*) (b) antibody at wound site relative to background (see Methods) in each genotype ((a) wild-type (n = 32), and *nub>bsk-RNAi* (n = 23); (b) wild-type (n = 28), and *nub>bsk-RNAi* (n = 22)). Mann-Whitney U-test; *** $p < 0.001$.

Supplementary Figure 3

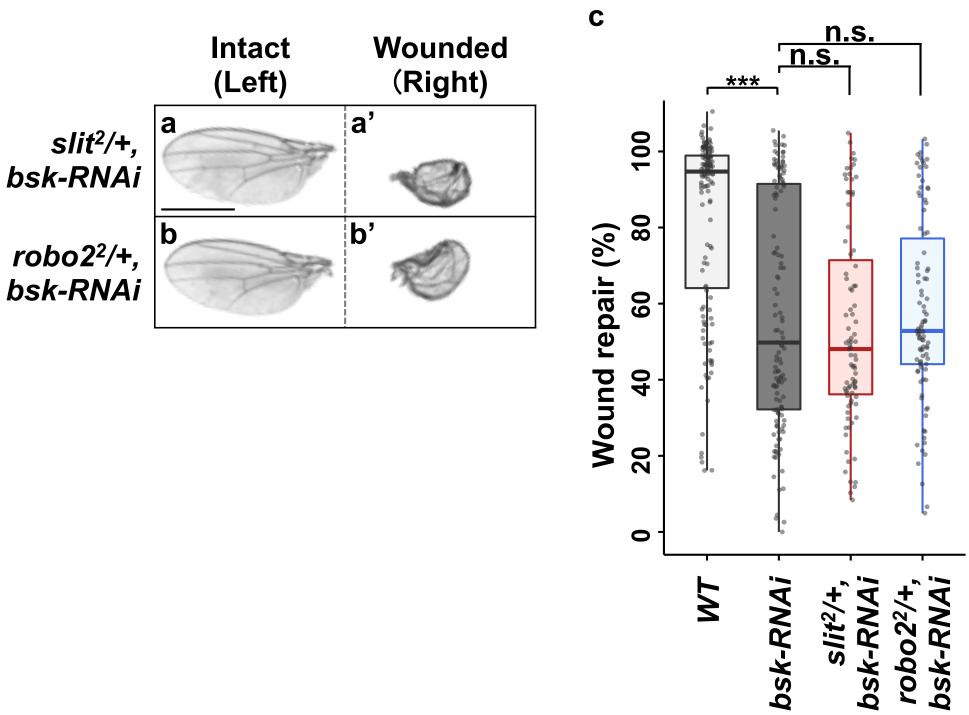

Blocking Slit-Robo2 does not exacerbate repair defect caused by *bsk-RNAi*.

(a-b') Control intact (a, b) and wounded (a', b') adult wings in each genotype taken from the same individuals. All pictures were taken at the same magnification. Scale bar, 500µm. (g) Boxplot with dots representing wound repair (%) (see Methods) in each genotype (wild-type (n = 136), *bsk-RNAi* (n = 131), *slit*^{2}*/+, bsk-RNAi* (n = 87), and *robo2*^{2}*/+, bsk-RNAi* (n = 94)). Mann-Whitney *U*-test; *** p < 0.001; n.s.; not significant.

Supplementary Figure 4

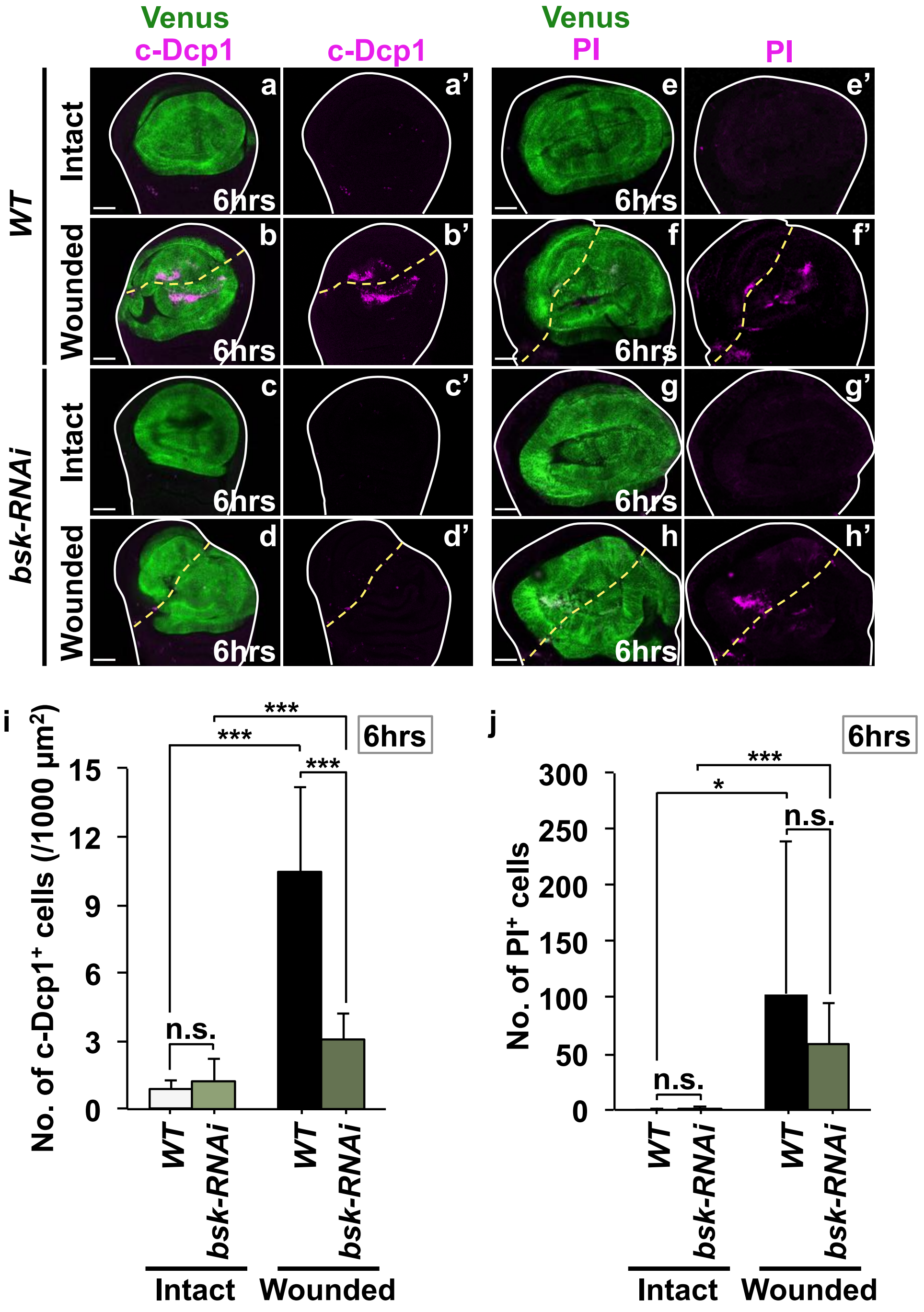

Physical wounding induces JNK-dependent apoptosis.

(a-h') Apoptotic cells were visualized with c-Dcp1 antibody staining (magenta) in the wing discs of wild-type (a-b') and *nub>bsk-RNAi* (c-d') larvae at 6hrs after wounding. Necrotic cells were detected by PI staining (magenta) in the wing discs of wild-type (e-f') and *nub>bsk-RNAi* (g-h') larvae at 6hrs after wounding. Wing pouches were

labeled with Venus using the *nub-gal4* driver (green). Yellow dashed lines indicate the positions of wounds. Scale bars, 50μm. (i) The number of apoptotic cells detected by c-Dcp1 antibody in a stacked *xy* cross-section of wing pouch at 6hrs after wounding in each genotype (wild-type (intact: n = 12, wounded: n = 11), *nub>bsk-RNAi* (intact: n = 20, wounded: n = 13)). (j) The number of necrotic cells detected by PI staining in a single *xy* cross-section of wing pouch at 6hrs after wounding in each genotype (wild-type (intact: n = 9, wounded: n = 8), *nub>bsk-RNAi* (intact: n = 10, wounded: n = 10)). Welch's T-test; mean ± s.d.; * $p < 0.05$, *** $p < 0.001$; n.s.; not significant.

Supplementary Figure 5

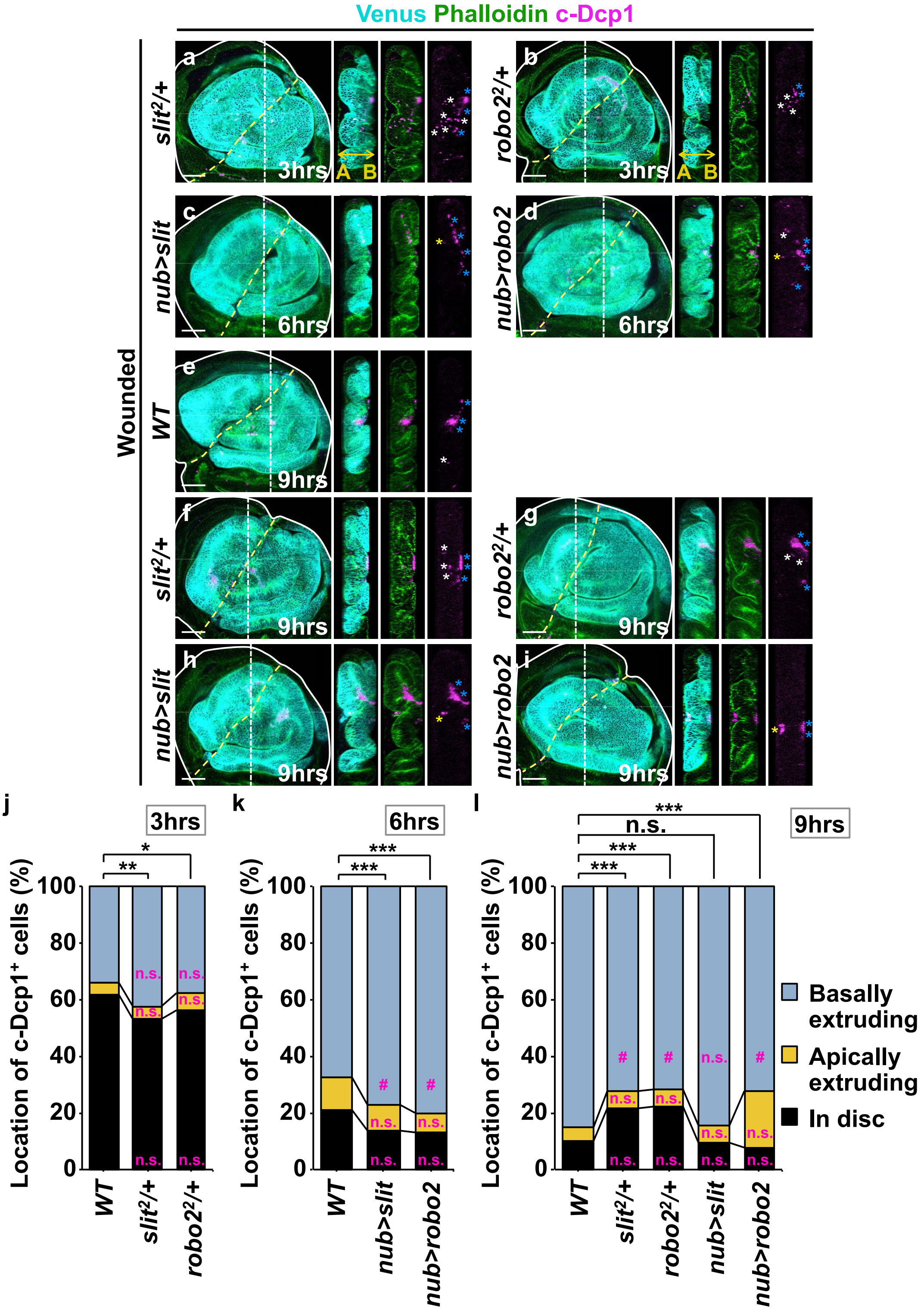

Extrusion of dying cells proceeds in a manner dependent on Slit-Robo2 signaling.

(a-i) Images show *xy* and *yz* cross-sections of wing discs of *slit*^{2}/+ (a), and *robo2*^{2}/+ (b)

larvae dissected at 3hrs after wounding; *xy* and *yz* cross-sections of *nub>slit* (c), and *nub>robo2* (d) larvae dissected at 6hrs after wounding; *xy* and *yz* cross-sections of wild-type (e), *slit*^{2}/+ (f), *robo2*^{2}/+ (g), *nub>slit* (h), and *nub>robo2* (i) larvae dissected at 9hrs after wounding. Dying cells were detected by anti-c-Dcp1 staining (magenta) and wing pouches were marked with Venus using the *nub-gal4* driver (cyan), and F-actin was visualized with Phalloidin (green). Yellow dashed lines indicate the positions of wounds. White dashed lines indicate the positions of *yz* cross-section shown in the right panel. The two-direction arrow indicates apical (A) and basal (B) sides of the disc. Asterisks in right panels indicate dying cells classified as “in disc” (white), “apically extruding” (yellow), and “basally extruding” (blue). Scale bars, 50µm. (j-l) Quantification of the ratio of dying cells classified into 3 types (as shown in Fig. 2a) at 3hrs after wounding in each genotype (j) (*slit*^{2}/+ ($n = 12$), and *robo2*^{2}/+ ($n = 13$)); 6hrs after wounding in each genotype (k) (*nub>slit* ($n = 13$), and *nub>robo2* ($n = 13$)); 6hrs after wounding in each genotype (l) (wild-type ($n = 12$), *slit*^{2}/+ ($n = 14$), *robo2*^{2}/+ ($n = 8$), *nub>slit* ($n = 10$), and *nub>robo2* ($n = 12$)). Chi-squared test; * $p < 0.05$, ** $p < 0.01$, *** $p < 0.001$; n.s.; not significant. # the absolute value of adjusted residual > 2.56. See Supplementary Information for detailed genotypes.

Supplementary Figure 6

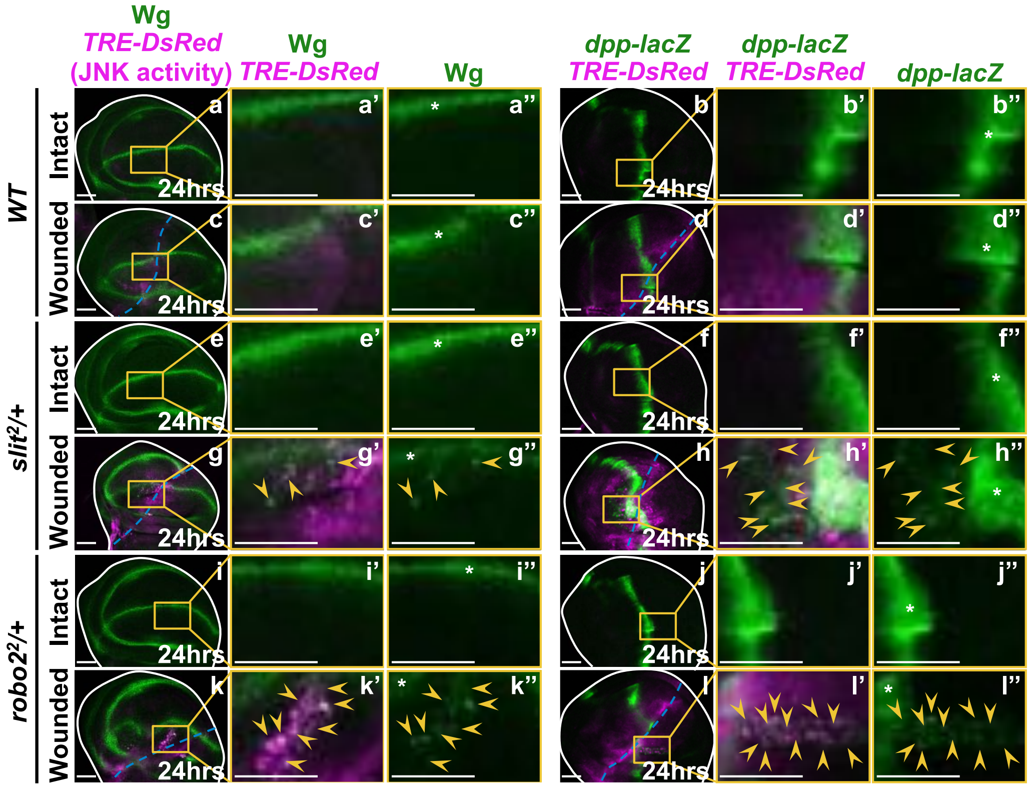

Aberrant Wg and Dpp express in or nearby JNK activation.

(a-l'') *xy* cross-section images of intact and wounded wing discs of wild-type (a-d''), *slit*^{2}/+ (e-h''), and *robo2*^{2}/+ (i-l'') larvae at 24hrs after wounding. JNK activity was detected with *TRE-DsRed* (magenta). Wg and Dpp expressions were detected using anti-Wg and anti-β-gal antibody (for *dpp-lacZ*), respectively (green). Blue dashed lines indicate wounds. Yellow arrowheads indicate representative cells expressing aberrantly high levels of Wg or Dpp with or nearby JNK activation. Asterisks indicate endogenous expression. Scale bars, 50μm.

Supplementary Figure 7

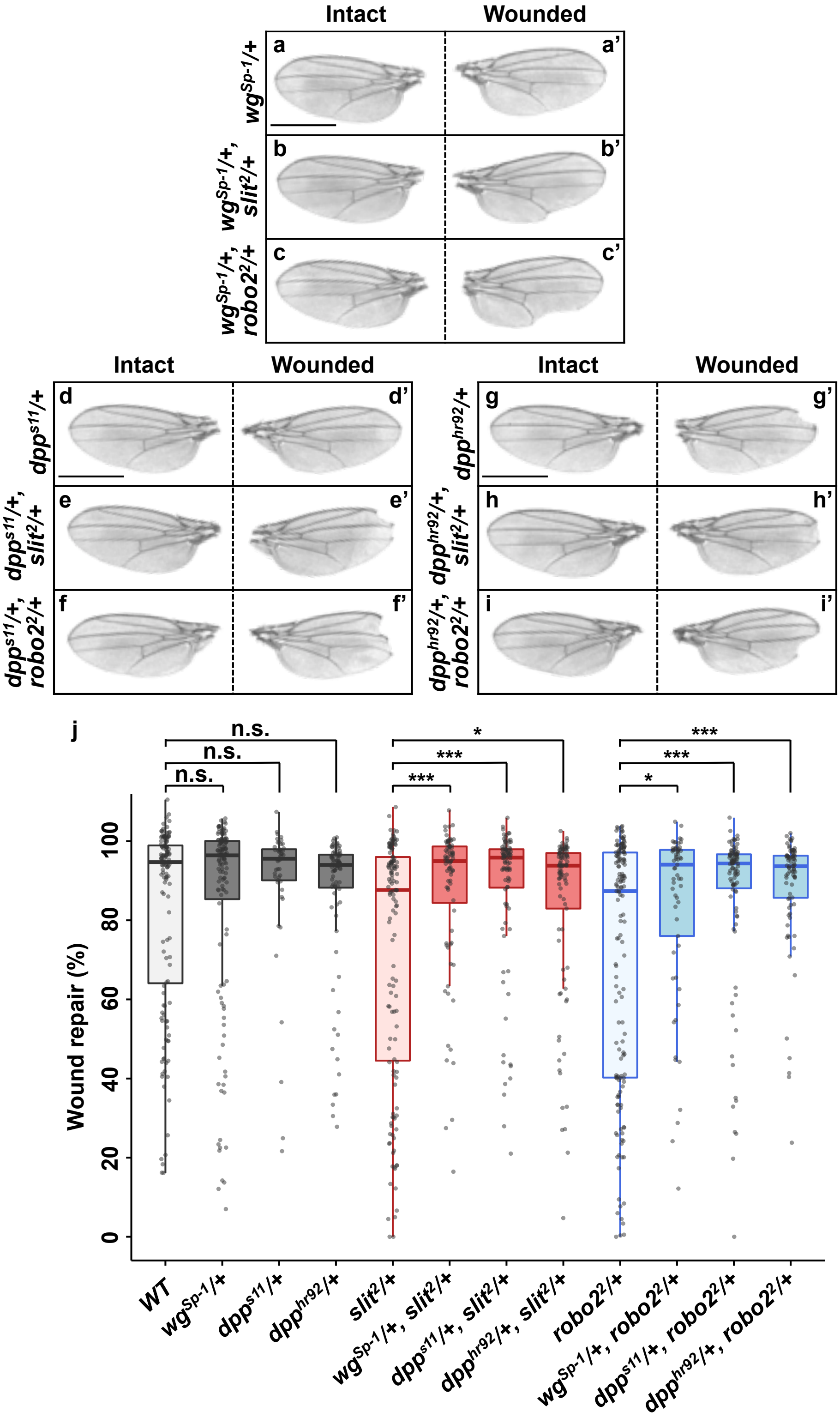

Other mutant alleles of *wg* and *dpp* also rescue the repair defect caused by Slit-Robo2 downregulation.

(a-i’) Control intact (a-i) and wounded (a’-i’) adult wings in each genotype taken from the same individuals after wounding. All pictures were taken at the same magnification. Scale bar, 500µm. (j) Boxplot with dots representing wound repair (%) (see Method for details) in each genotype (wild-type (n = 138), $wg^{Sp\text{-}1}$/+ (n = 128), dpp^{s11}/+ (n = 48), dpp^{hr92}/+ (n = 84), $slit^{2}$/+ (n = 139), $slit^{2}$/+, $wg^{Sp\text{-}1}$/+ (n = 81), $slit^{2}$/+, dpp^{s11}/+ (n = 92), $slit^{2}$/+, dpp^{hr92}/+ (n = 93), $robo2^{2}$/+ (n = 154), $robo2^{2}$/+, $wg^{Sp\text{-}1}$/+ (n = 61), $robo2^{2}$/+, dpp^{s11}/+ (n = 98), and $robo2^{2}$/+, dpp^{hr92}/+ (n = 83)). Mann-Whitney *U*-test; * $p < 0.05$, *** $p < 0.001$; n.s.; not significant. See Supplementary Information for detailed genotypes.

Supplementary Figure 8

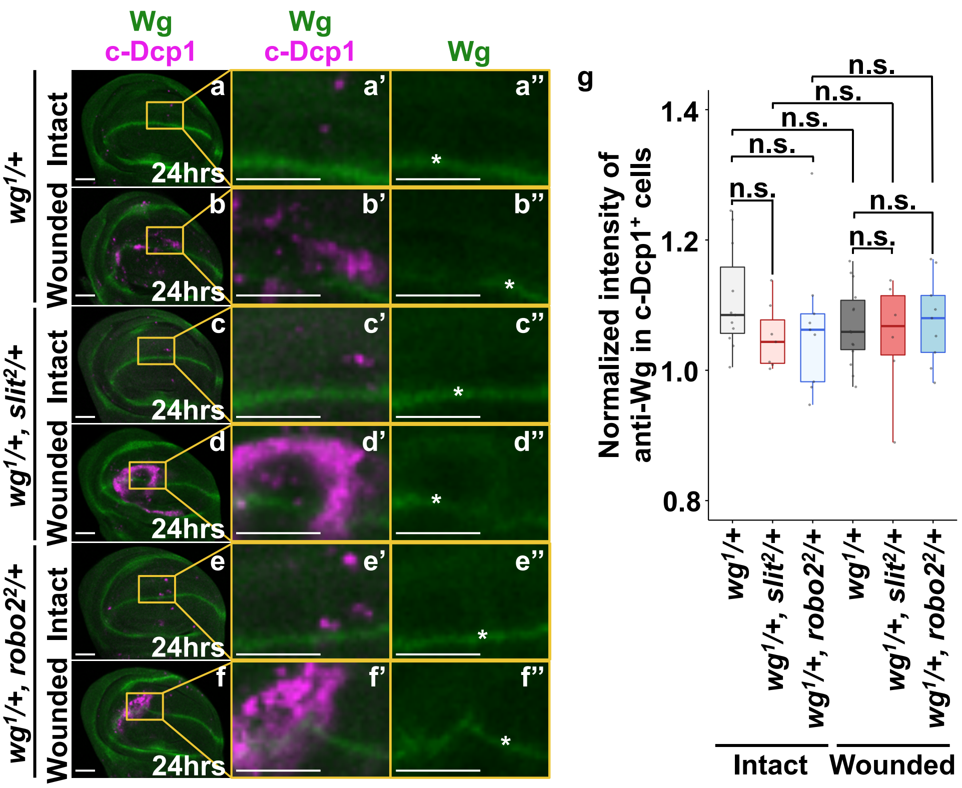

***wg*[1]/+ suppresses the emergence of aberrant cells with excess production of Wg.**

(a-f'') *xy* cross-section images of intact and wounded wing discs of *wg*[1]/+ (a-b''), *wg*[1]/+, *slit*[2]/+ (c-d''), and *wg*[1]/+, *robo2*[2]/+ (e-f'') larvae at 24hrs after wounding. *wg*[1] has a small deletion in the enhancer region of *wg* gene. Dying cells ware detected with c-Dcp1 antibody (magenta). Wg expression was detected using anti-Wg antibody (green). Asterisks indicate endogenous expression. Scale bars, 50µm. (g) Boxplot with dots representing normalized intensity of anti-Wg in c-Dcp1-positive cells (see Methods) in each genotype (*wg*[1]/+ (intact: n = 11, wounded: n = 14), *wg*[1]/+, *slit*[2]/+ (intact: n = 7, wounded: n = 6), and *wg*[1]/+, *robo2*[2]/+ (intact: n = 9, wounded: n = 9)). Mann-Whitney *U*-test; n.s.; not significant.

Detailed Genotype

Figure 1

a, a’ *nub-gal4 / +; UAS-CD8-PARP-Venus / +*

b, b’ *nub-gal4 / +; UAS-CD8-PARP-Venus / UAS-bsk-RNAi*

c, c’ *nub-gal4 / slit*^{2}*; UAS-CD8-PARP-Venus / +*

d, d’ *nub-gal4 / +; UAS-CD8-PARP-Venus / UAS-slit-RNAi*

e, e’ *nub-gal4 / robo2*^{2}*; UAS-CD8-PARP-Venus / +*

f, f’ *nub-gal4 / +; UAS-CD8-PARP-Venus / UAS-robo2-RNAi*

g From left to right

nub-gal4 / +; UAS-CD8-PARP-Venus / +

nub-gal4 / +; UAS-CD8-PARP-Venus / UAS-bsk-RNAi

nub-gal4 / slit^{2}*; UAS-CD8-PARP-Venus / +*

nub-gal4 / +; UAS-CD8-PARP-Venus / UAS-slit-RNAi

nub-gal4 / robo2^{2}*; UAS-CD8-PARP-Venus / +*

nub-gal4 / +; UAS-CD8-PARP-Venus / UAS-robo2-RNAi

h-i’’’ *nub-gal4, UAS-GFP*^{S65T} */ slit-lacZ; + / +*

j-k’’’ *nub-gal4, UAS-GFP*^{S65T} */ slit-lacZ; UAS-bsk-RNAi / +*

Figure 2

b *nub-gal4 / +; UAS-CD8-PARP-Venus / +*

c *nub-gal4 / slit*^{2}*; UAS-CD8-PARP-Venus / +*

d *nub-gal4 / robo2*^{2}*; UAS-CD8-PARP-Venus / +*

e, f From left to right

nub-gal4 / +; UAS-CD8-PARP-Venus / +

nub-gal4 / slit^{2}*; UAS-CD8-PARP-Venus / +*

	nub-gal4 / robo2^{2}*; UAS-CD8-PARP-Venus / +*
g	*nub-gal4 / +; UAS-CD8-PARP-Venus / +*
h	*nub-gal4 / +; UAS-CD8-PARP-Venus / UAS-slit*
i	*nub-gal4 / +; UAS-CD8-PARP-Venus / UAS-robo2*
j, k	From left to right
	nub-gal4 / +; UAS-CD8-PARP-Venus / +
	nub-gal4 / +; UAS-CD8-PARP-Venus / UAS-slit
	nub-gal4 / +; UAS-CD8-PARP-Venus / UAS-robo2

Figure 3

a-d''	*+ / +; rn-gal4, UAS-CD8-PARP-Venus / +*
e-h''	*+ / slit*^{2}*; rn-gal4, UAS-CD8-PARP-Venus / +*
i-l''	*+ / robo2*^{2}*; rn-gal4, UAS-CD8-PARP-Venus / +*
m, n	From left to right
	+ / +; rn-gal4, UAS-CD8-PARP-Venus / +
	+ / slit^{2}*; rn-gal4, UAS-CD8-PARP-Venus / +*
	+ / robo2^{2}*; rn-gal4, UAS-CD8-PARP-Venus / +*
	+ / +; rn-gal4, UAS-CD8-PARP-Venus / +
	+ / slit^{2}*; rn-gal4, UAS-CD8-PARP-Venus / +*
	+ / robo2^{2}*; rn-gal4, UAS-CD8-PARP-Venus / +*

Figure 4

a, a'	*wg*^{1} */ +; rn-gal4, UAS-CD8-PARP-Venus / +*
b, b'	*wg*^{1} */ slit*^{2}*; rn-gal4, UAS-CD8-PARP-Venus / +*
c, c'	*wg*^{1} */ robo2*^{2}*; rn-gal4, UAS-CD8-PARP-Venus / +*
d, d'	*dpp*^{d6} */ +; rn-gal4, UAS-CD8-PARP-Venus / +*
e, e'	*dpp*^{d6} */ slit*^{2}*; rn-gal4, UAS-CD8-PARP-Venus / +*
f, f'	*dpp*^{d6} */ robo2*^{2}*; rn-gal4, UAS-CD8-PARP-Venus / +*

g From Left to right

nub-gal4 / +; UAS-CD8-PARP-Venus / +

wg^{1} */ +; rn-gal4, UAS-CD8-PARP-Venus / +*

dpp^{d6} */ +; rn-gal4, UAS-CD8-PARP-Venus / +*

nub-gal4 / slit^{2}*; UAS-CD8-PARP-Venus / +*

wg^{1} */ slit*^{2}*; rn-gal4, UAS-CD8-PARP-Venus / +*

dpp^{d6} */ slit*^{2}*; rn-gal4, UAS-CD8-PARP-Venus / +*

nub-gal4 / robo2^{2}*; UAS-CD8-PARP-Venus / +*

wg^{1} */ robo2*^{2}*; rn-gal4, UAS-CD8-PARP-Venus / +*

dpp^{d6} */ robo2*^{2}*; rn-gal4, UAS-CD8-PARP-Venus / +*

Supplementary Figure 1

b-d *nub-gal4 / +; UAS-CD8-PARP-Venus / +*

e *nub-gal4 / slit*^{2}*; UAS-CD8-PARP-Venus / +*

f *nub-gal4 / robo2*^{2}*; UAS-CD8-PARP-Venus / +*

Supplementary Figure 2

a, b From left to right

nub-gal4, UAS-GFP^{S65T} */ slit-lacZ; + / +*

nub-gal4, UAS-GFP^{S65T} */ slit-lacZ; UAS-bsk-RNAi / +*

Supplementary Figure 3

a, a’ *nub-gal4 / slit*^{2}*; UAS-CD8-PARP-Venus / UAS-bsk-RNAi*

b, b’ *nub-gal4 / robo2*^{2}*; UAS-CD8-PARP-Venus / UAS-bsk-RNAi*

c From Left to right

nub-gal4 / +; UAS-CD8-PARP-Venus / +

nub-gal4 / +; UAS-CD8-PARP-Venus / UAS-bsk-RNAi

nub-gal4 / slit^{2}*; UAS-CD8-PARP-Venus / UAS-bsk-RNAi*

nub-gal4 / robo2^{2}*; UAS-CD8-PARP-Venus / UAS-bsk-RNAi*

Supplementary Figure 4

a-b’ *nub-gal4 / +; UAS-CD8-PARP-Venus / +*

c-d’ *nub-gal4 / +; UAS-CD8-PARP-Venus / UAS-bsk-RNAi*

e-f’ *nub-gal4 / +; UAS-CD8-PARP-Venus / +*

g-h’ *nub-gal4 / +; UAS-CD8-PARP-Venus / UAS-bsk-RNAi*

i, j From Left to right

nub-gal4 / +; UAS-CD8-PARP-Venus / +

nub-gal4 / +; UAS-CD8-PARP-Venus / UAS-bsk-RNAi

nub-gal4 / +; UAS-CD8-PARP-Venus / +

nub-gal4 / +; UAS-CD8-PARP-Venus / UAS-bsk-RNAi

Supplementary Figure 5

a *nub-gal4 / slit*^{2}*; UAS-CD8-PARP-Venus / +*

b *nub-gal4 / robo2*^{2}*; UAS-CD8-PARP-Venus / +*

c *nub-gal4 / +; UAS-CD8-PARP-Venus / UAS-slit*

d *nub-gal4 / +; UAS-CD8-PARP-Venus / UAS-robo2*

e *nub-gal4 / +; UAS-CD8-PARP-Venus / +*

f *nub-gal4 / slit*^{2}*; UAS-CD8-PARP-Venus / +*

g *nub-gal4 / robo2*^{2}*; UAS-CD8-PARP-Venus / +*

h *nub-gal4 / +; UAS-CD8-PARP-Venus / UAS-slit*

i *nub-gal4 / +; UAS-CD8-PARP-Venus / UAS-robo2*

j From left to right

nub-gal4 / +; UAS-CD8-PARP-Venus / +

nub-gal4 / slit^{2}*; UAS-CD8-PARP-Venus / +*

nub-gal4 / robo2^{2}*; UAS-CD8-PARP-Venus / +*

k From left to right

nub-gal4 / +; UAS-CD8-PARP-Venus / +

nub-gal4 / +; UAS-CD8-PARP-Venus / UAS-slit

nub-gal4 / +; UAS-CD8-PARP-Venus / UAS-robo2

l From left to right

nub-gal4 / +; UAS-CD8-PARP-Venus / +

nub-gal4 / slit^{2}*; UAS-CD8-PARP-Venus / +*

nub-gal4 / robo2^{2}*; UAS-CD8-PARP-Venus / +*

nub-gal4 / +; UAS-CD8-PARP-Venus / UAS-slit

nub-gal4 / +; UAS-CD8-PARP-Venus / UAS-robo2

Supplementary Figure 6

a-d'' *TRE-DsRed / +; rn-gal4, UAS-CD8-PARP-Venus / +*

e-h'' *TRE-DsRed / slit*^{2}*; rn-gal4, UAS-CD8-PARP-Venus / +*

g-l'' *TRE-DsRed / robo2*^{2}*; rn-gal4, UAS-CD8-PARP-Venus / +*

Supplementary Figure 7

a, a' *wg*$^{Sp-1}$ */ +; rn-gal4, UAS-CD8-PARP-Venus / +*

b, b' *wg*$^{Sp-1}$ */ slit*^{2}*; rn-gal4, UAS-CD8-PARP-Venus / +*

c, c' *wg*$^{Sp-1}$ */ robo2*^{2}*; rn-gal4, UAS-CD8-PARP-Venus / +*

d, d' *dpp*^{s11} */ +; rn-gal4, UAS-CD8-PARP-Venus / +*

e, e' *dpp*^{s11} */ slit*^{2}*; rn-gal4, UAS-CD8-PARP-Venus / +*

f, f' *dpp*^{s11} */ robo2*^{2}*; rn-gal4, UAS-CD8-PARP-Venus / +*

g, g' *dpp*^{hr92} */ +; rn-gal4, UAS-CD8-PARP-Venus / +*

h, h' *dpp*^{hr92} */ slit*^{2}*; rn-gal4, UAS-CD8-PARP-Venus / +*

i, i' *dpp*^{hr92} */ robo2*^{2}*; rn-gal4, UAS-CD8-PARP-Venus / +*

j From Left to right

nub-gal4 / +; UAS-CD8-PARP-Venus / +

wg^{Sp-1} */ +; rn-gal4, UAS-CD8-PARP-Venus / +*

dpp^{s11} */ +; rn-gal4, UAS-CD8-PARP-Venus / +*

dpp^{hr92} */ +; rn-gal4, UAS-CD8-PARP-Venus / +*

nub-gal4 / slit^{2}*; UAS-CD8-PARP-Venus / +*

wg^{Sp-1} */ slit*^{2}*; rn-gal4, UAS-CD8-PARP-Venus / +*

dpp^{s11} */ slit*^{2}*; rn-gal4, UAS-CD8-PARP-Venus / +*

dpp^{hr92} */ slit*^{2}*; rn-gal4, UAS-CD8-PARP-Venus / +*

nub-gal4 / robo2^{2}*; UAS-CD8-PARP-Venus / +*

wg^{Sp-1} */ robo2*^{2}*; rn-gal4, UAS-CD8-PARP-Venus / +*

dpp^{s11} */ robo2*^{2}*; rn-gal4, UAS-CD8-PARP-Venus / +*

dpp^{hr92} */ robo2*^{2}*; rn-gal4, UAS-CD8-PARP-Venus / +*

Supplementary Figure 8

a-b’’ *wg*^{1} */ +; rn-gal4, UAS-CD8-PARP-Venus / +*

c-d’’ *wg*^{1} */ slit*^{2}*; rn-gal4, UAS-CD8-PARP-Venus / +*

e-f’’ *wg*^{1} */ robo2*^{2}*; rn-gal4, UAS-CD8-PARP-Venus / +*

g From Left to right

wg^{1} */ +; rn-gal4, UAS-CD8-PARP-Venus / +*

wg^{1} */ slit*^{2}*; rn-gal4, UAS-CD8-PARP-Venus / +*

wg^{1} */ robo2*^{2}*; rn-gal4, UAS-CD8-PARP-Venus / +*

wg^{1} */ +; rn-gal4, UAS-CD8-PARP-Venus / +*

wg^{1} */ slit*^{2}*; rn-gal4, UAS-CD8-PARP-Venus / +*

wg^{1} */ robo2*^{2}*; rn-gal4, UAS-CD8-PARP-Venus / +*
